# Selenium in Bone Health: Roles in Antioxidant Protection and Cell Proliferation 

**DOI:** 10.3390/nu5010097

**Published:** 2013-01-10

**Authors:** Huawei Zeng, Jay J. Cao, Gerald F. Combs

**Affiliations:** Grand Forks Human Nutrition Research Center, Agricultural Research Service, United States Department of Agriculture, Grand Forks, ND 58202, USA; E-Mails: jay.cao@ars.usda.gov (J.J.C.); gerald.combs@ars.usda.gov (G.F.C.)

**Keywords:** antioxidant, bone health, cell proliferation

## Abstract

Selenium (Se) is an essential trace element for humans and animals, and several findings suggest that dietary Se intake may be necessary for bone health. Such findings may relate to roles of Se in antioxidant protection, enhanced immune surveillance and modulation of cell proliferation. Elucidation of the mechanisms by which Se supports these cellular processes can lead to a better understanding of the role of this nutrient in normal bone metabolism. This article reviews the current knowledge concerning the molecular functions of Se relevant to bone health.

## 1. Introduction

Selenium (Se) is an essential nutrient, being required at 0.1–0.2 ppm in animal diets, and with a recommended daily allowance of 55 μg/day for both men and women [1]. It plays critical roles in a variety of physiological processes as an essential constituent of some 25 selenoenzymes in which it is present as the selenoamino acid, selenocysteine, not found in other proteins [2,3]. The daily needs for Se are met by almost all Americans [4]; however, certain populations in Europe, Africa and Asia have intakes much less than 25 μg/day [5]. 

It has been reported that Se inadequacy can retard growth and change bone metabolism [6,7]. Blood Se concentration has been found to be inversely related to the rate of bone turnover and positively correlated with the prevalence of low bone mineral density (BMD) in humans [8]. Low Se intakes have been associated with increased risk to bone disease [5,7,9,10]. The results of mechanistic studies have suggested an underlying mechanism, *i.e.*, that of Se playing an essential role in antioxidant defense supporting immune surveillance and cell proliferation and differentiation [3,7,10,11]. In this review, we summarize current knowledge of the effects of Se on bone and the underlying mechanisms.

## 2. Relationships of Se and Bone Metabolism

At least nine selenoproteins are known to be expressed in human fetal osteoblasts [12,13,14]. Their expression by bone cells would appear to contribute to protection against oxidative stress in the microenvironment of the bone. Excessive intracellular reactive oxygen species (ROS) levels are thought to contribute to the development of osteoporosis by inhibiting osteoblastic differentiation of bone marrow stromal cells (BMSCs) [15,16,17]. BMSCs cultured in the presence of low Se concentrations (5–10 nM) show reduced expression of glutathione peroxidases (GPXs), thioredoxin reductases (TRRs) and other selenoproteins, as well as the appearance of micronuclei indicative of chromosomal damage [18]. These effects were reversed by treatment with selenite (100 nM) [18]. Genetic data showed that a single nucleotide polymorphism (SNP) at codon 198 of GPX1, is associated with low bone mineral density (BMD), increased bone turnover markers [19] and Kaschin-Beck chondrodystrophy [20]. In addition, three SNPs in the selenoprotein S gene revealed four common haplotypes, showed a significant association to inflammatory signaling (e.g., cytokines), and has been found to be related to osteoarthritis risk [21].

Animal studies have shown Se-deprivation to change bone metabolism [6,7]. Such effects were associated with reductions in the activity of GPX1 and circulating concentrations of pituitary growth hormone, plasma insulin-like growth factor I and calcium, increases in circulating concentrations of parathyroid hormone, 1,25-dihydroxyvitamin D_3_, and increases in urinary calcium concentration [6,7]. That these changes were associated with reduced BMD and bone volume, and with impaired bone microarchitecture indicates they were associated with increased bone resorption [6,7]. Evidence suggests that a role of ROS in promoting bone resorption, as osteoporotic bone tissue, when compared to non-osteoporotic tissue, showed greater levels of lipid peroxides and H_2_O_2_, but lower activities of the antioxidant enzymes superoxide dismutase, GPX3 and glutathione-*S*-transferase [22]. Second-generation of Se-deprived rats also showed reduced growth, and osteopenia [6,23]. Studies with a transgenic mouse model with an osteochondroprogenitor-specific deletion of the selenocysteine tRNA gene (essential for selenoprotein synthesis) [24] showed that the preservation of selenoprotein activity in osteochondroprogenitors is essential to skeletogenesis and the maintenance of cartilage viability. This finding may explain growth retardation, epiphyseal growth plate abnormalities, delayed skeletal ossification and marked chondronecrosis of articular cartilage observed in this model [24]. 

Studies in humans provide evidence that Se status can be related to bone health. Plasma Se concentration was found to be inversely related to the rate of bone turnover and positively correlated with the prevalence of BMD in healthy euthyroid post-menopausal women [8]. Several years ago, low Se status has been reported in Germany in a group of pediatric patients fed low-Se formula diets; these patients also had low BMD [25,26]. Similarly, low BMD and osteoarthropathy have been identified in residents of Tibet where Se contents of soils are low [25,26,27]. A case-control study found Se intake to be inversely associated reduced risk of osteoporotic hip fracture in an elderly population of smokers [10]. 

## 3. Mechanisms of Se in Supporting Bone Health

There are only a few reported studies of effects of Se on in bone cells [28,29,30]. However, it is likely that the cellular mechanisms observed in other cell types also occur in bone cells. 

### 3.1. Se Metabolism

**Figure 1:**
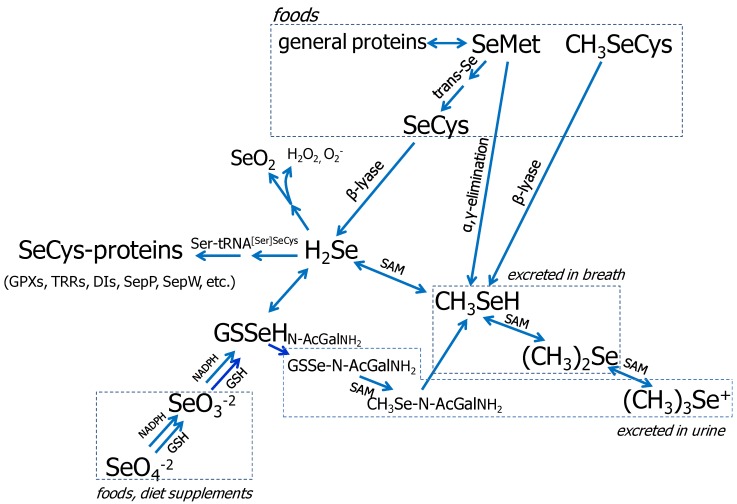
Metabolism of biologically important Se-compounds. Note: This figure is adapted with permission from [38], Copyright © 2005 Margaret P. Rayman; and from [39], Copyright © 2004 Cancer Research UK.

Foods contain various amounts and chemical forms of Se, most of which is covalently bound to carbon in organic molecules including as selenomethionine (SeMet), selenocysteine (SeCys) and Se-methylselenocysteine (SeMSC) [31,32]. These forms can each be metabolized to selenide (H_2_Se), which serves as the obligate intermediate in selenoprotein synthesis (Figure 1) [33,34,35,36,37]. SeMet, unlike other forms of Se, can substitute for methionine (Met) in protein synthesis for which reason it is incorporated non-specifically into proteins [38,39]. Selenide can also be metabolized by methylation and sugar-derivation to produce a variety of excreted products (Figure 1) [36]. These include selenosugars, which are the major urinary metabolites in humans, and methylselenol (CH_3_SeH), which is regarded as the major anticarcinogenic Se metabolite [40].

**Figure 2:**
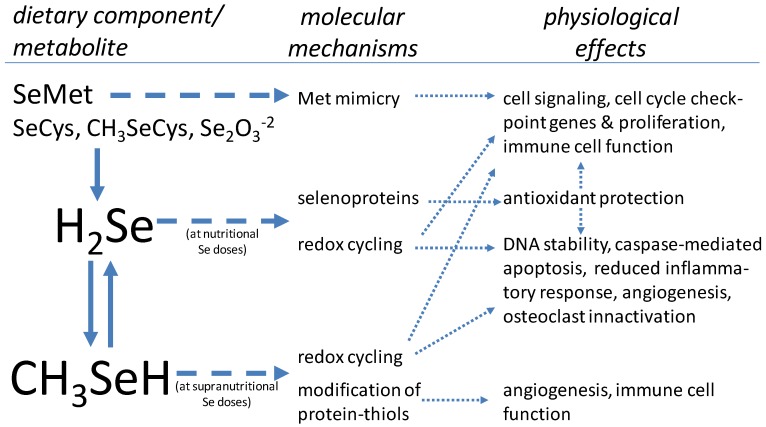
Molecular mechanisms of Se related to bone health [40,41,42,43,44,45].

The Se-species that are likely to be present in cells (H_2_Se, CH_3_SeH and SeMet) may be biologically active, executing effects by several means: producing ROS by redox cycling of H_2_Se and CH_3_SeH; modification of protein-thiols by CH_3_SeH; and mimicking Met in protein synthesis by SeMet (Figure 2). It has been suggested that these activities are underlying the cellular effect of high doses of Se [41,42], which includes the regulation of cell cycle and apoptosis. Although many of these biochemical pathways have been characterized in several different cell types, these molecular events may also occur in bone cells.

### 3.2. The Selenoproteins

Only 25 selenoprotein genes have been identified in the human genome (24 in the mouse) [3,46]. These include the GPXs, TRRs, iodothyronine deiodinases (DIs), selenprotein P (SEPP1) and several less well characterized selenoproteins [3,46]. Their synthesis involves the co-translational biosynthesis of SeCys by a unique process in which the UGA codon is recoded to specify SeCys incorporation instead of translational stop [35]. Selenoprotein biosynthesis is dependent on dietary Se intake, genotype and inflammatory tone [3,35,46,47]. At limiting levels, this is thought to result in a hierarchal pattern of selenoprotein expression correlating with the relative significance of selenoproteins in cellular function [3,46]. The nutritional roles of Se are thought to be discharged by these selenoproteins most of which appear to exhibit oxidoreductase activities [3,46]. Accordingly, these selenoproteins have been shown to protect cells from ROS, reactive nitrogen species and oxidative damage [47].

### 3.3. Se in Protection from Oxidative Stress

Inadequate Se intake can result in increased levels of ROS/oxidative stress, particularly in individuals of low status with respect to other antioxidants (e.g., vitamins E and C) [48]. All of the enzymatically characterized seleoproteins catalyze not only redox reactions at sulfhydryl groups/disulfides, but also C–I bonds catalyzed by deiodinases or S–O bonds catalysed by MetSulfoxide Reductase B. [47,48]. An increase in oxidative stress, achieved experimentally either by elevating intracellular ROS or adding exogenous H_2_O_2 _ at micromolar concentrations, has been shown to inhibit growth in a wide variety of mammalian cells [49]. Many studies have established that ROS-related oxidative stress induces cell cycle arrest, senescence, apoptosis and/or necrotic cell death [49,50].

ROS have been shown to be important signaling molecules at submicomolar levels, and a biphasic effect has been demonstrated on cellular proliferation with ROS—especially hydrogen peroxide and superoxide—in which low levels (usually submicromolar concentrations) induce growth but higher concentrations (usually >10–30 μM) induce apoptosis or necrosis [49,50]. For example, cell surface receptors produce ROS upon activation; this includes receptors for epidermal growth factor, platelet-derived growth factor, insulin-like growth factor, vascular endothelial growth factor and various cytokines [47,51,52]. At higher intracellular levels, ROS are damaging to organelles, particularly mitochondria [53], which may result in energy depletion, accumulation of cytotoxic mediators and cell death [53]. Thus, compromised oxidative defense may predispose tissues to damage by environmental stress factors. The maintenance of intracellular redox homeostasis is dependent on a complex web of antioxidant factors including both low molecular weight molecules (e.g., glutathione and thioredoxin) and protein antioxidants (e.g., certain selenoproteins) [53]. 

### 3.4. Se in Cell Proliferation/Differentiation

Under normal physiological conditions, most cells appear to require Se for normal growth because of their need of selenoproteins. For example, 50 nmol/L Se, as either selenite or SeMet, is required for the growth of human fibroblasts, lymphocytes and Chinese hamster ovary cells [54,55,56]. Selenium plays a critical role in cell cycle progression; its omission results in G2 cell cycle arrest [56]. Studies have shown that treatment with either selenite or SeMet can up-regulate the expression of several cell cycle-related genes: *c-Myc*, *cyclin C*, proliferating cell nuclear antigen, cyclin-dependent kinase (*cdk*)*1*, *cdk2*, *cdk4*, *cyclin B* and *cyclin D2* mRNA [56]. In addition, Se increased total cellular phosphorylated proteins [56]. These observations are consistent with the finding that the up-regulation of *cdk1*, *cdk2*, *cdk4*, *cyclin B* and *cyclin D2* led to the promotion of cell cycle progression, particularly G2/M transition and/or the reduction of apoptosis, *in vivo* and *in vitro* [42,57,58]. A few cases have been reported in which certain cells can survive in the absence of Se [59]. This includes most hepatocellular carcinoma cell lines [59]. We found [60] that Se-deprivation did not affect cell cycle progress and apoptosis in human colon Caco-2 cells even though GPX activity was limited to 8% of that of controls. In that model, Se treatment increased the expression of humoral defense and tumor suppressor-related genes while decreasing the expression of pro-inflammatory genes [60]. 

Some, but not all, studies have found immune cell proliferation to be sensitive to Se-deprivation [11,56]. For example, when derived immune T and B cells (e.g., Jurkat and HL60 cells) were cultured in a Se-deficient, serum-free medium, GPX and TRR activities were low and cells did not survive [11,56]. That Se-deprivation increased ROS production in these cells was indicated by the fact that treatment with a lipid soluble radical-scavenging antioxidant (vitamin E) restored survival without affecting selenoenzyme expression [11]. Selenium-deficient lymphocytes are less able to proliferate in response to a mitogen, but the response can be improved by supplementing with Se [61].

Adequate Se status has been shown to be essential for an optimum immune response, affecting both the innate and acquired immunity [62]. Inadequate Se intake has been associated with low titers of IgG and IgM [63]. Redox tone is known to play a role in modulating the activation of T cells to effectors [61], and the absence of selenoproteins in T cells led to decreased pools of mature T cells and defective T cell activation [64]. 

Only a few Studies have addressed mechanisms underlying the relationship of Se status and bone cell proliferation. However, it is likely that the cellular mechanisms described in other cell types may also occur in bone cells. Indeed, selenoproteins are expressed in osteoblastic cells, and Se supplement restores the antioxidative capacity and prevents cell damage in bone cells (e.g., BMSCs) [12,13,14,18]. For example, TRR has been found to be the responsive gene of 1-α,25(OH)_2_-vitamin D_3_, the hormone that stimulates bone cell growth and differentiation [65]. 

Normal bone remodeling appears to depend on the controlled function of ROS. Remodeling is a lifelong process involving the removal of old and damaged bone matrix by osteoclasts and replacement with new bone formed by osteoblasts [66]. Osteoclasts are bone-specific multi-nucleated cells generated from hematopoietic monocyte precursor cells [67]; this differentiation is regulated through both receptor-activation of nuclear factor κB ligand (RANKL) and macrophage-colony stimulating factor (M-CSF) [66,67]. Bone loss, regardless of cause, reflects increased function of osteoclasts relative to that of osteoblasts [66,67]. The latter bone cells generate superoxide by NADPH oxidase [68], and intracellular ROS is known to stimulate the differentiation and formation of mature osteoclasts [69]. That Se and other antioxidants may have a role in this process is suggested by the finding that Se-treatment suppressed RANKL-induced gene expression and phosphor-IκBα activtion [70,71,72] to reduce RANKL-mediated ROS generation and inhibit the signaling of osteoclast differentiation. 

Adequate Se intake appears to play an essential role in osteoclast/osteoblast cell proliferation and differentiation by the regulation of ROS status. Signaling by extracellular signal-regulated kinases ERK1/2 is known to mediate the inhibitory effect of H_2_O_2_ on osteoblastic differentiation in rabbit BMSCs and MC3T3-E1 preosteoblastic cells [15,73].

Recent findings indicate that Se-treatment can protect BMSCs against H_2_O_2_-induced inhibition of osteoblastic differentiation by inhibiting oxidative stress and ERK activation [74]. Treatment with nanomolar amounts of Se, which increased GSH concentraitions and GPX1 expression, also enhanced the expression of type I collagen and alkaline phosphatase, and the deposition of calcium in rat marrow stromal cells (MSCs) [74]. These observations suggest that, at the cellular level, Se was capable of enhancing osteoblastic differentiation of MSCs by reducing basal oxidative stress [74]. In contrast, when selenoprotein expression was decreased due to an inadequate Se intake, ROS levels and phosphorylation status remained elevated and contributed to pathologically exacerbated signaling and enhanced osteoclast activity [13]. Because osteoblasts express several selenoproteins (e.g., GPX, SelP) at high levels it would follow that adequate Se intake is necessary to support this system of osteoblast antioxidative defense that may be relevant for the protection against ROS produced by osteoclasts during bone remodeling [13]. 

### 3.5. Effects of High Doses of Se

Cells treated with high levels of Se typically show alterations in proliferation and differentiation. While these effects have been found to be strongest in cancer cells (and would appear to contribute to the anticarcinogenic activity of Se), they also occur in noncancerous cells [75]. Cells treated with high levels of selenite typically show arrested at the S/G2-M phases with an increase in cdk2 kinase activity and DNA damage-inducible *gadd* gene [76,77,78,79,80]. In contrast, the metabolite methylselenol (CH_3_SeH) and its metabolic precursors have been found to induce caspase-mediated apoptosis in those cells [81,82]. Methylselenol has also been shown to activate the caspase cascade and apoptosis [76,77,83]. Submicromolar levels of CH_3_SeH have been found to cause cell cycle arrest, leading to increases in cells in the G1 and G2 phases with concomitant reductions of cells in the S-phase [75,76,77,84]. A metabolic precursor of CH_3_SeH, methylselenocyanate (SeMSC), has been shown to exert moderate anti-proliferative effects through G1 cell cycle arrest; whereas, selenite rapidly blocked DNA synthesis and arrested cells in the S phase [76,77,84]. Cells exposed to methylselenocysteine (CH_3_SeCys) were arrested at the G1 phase of the cell cycle as there was a decrease in the cdk2 kinase activity accompanied by a decrease in cyclin E-cdk2 content [78]. In addition, CH_3_SeH exposure of noncancerous NCM460 colon cells inhibited cell growth and led to increases in cells in both the G1 and G2 phases with a concomitant reduction of cells in S-phase and an induction of apoptosis [75]. These responses were associated with reduced phosphorylation of ERK1/2, p38 mitogen-activated protein kinase (MAPK) and cellular myelocytomatosis oncogene (c-Myc) and up-regulation of phosphorylation of sarcoma and focal adhesion kinase survival signals [75]. In general, the doses of Se of these *in vitro* findings may be higher than that of the *in vivo* findings (human studies) [75,85,86,87]. However, the underlying biochemical mechanisms are likely to be the same.

High-dose Se treatment appears to enhance the capacity of lymphocytes to respond to mitogen or alloantigen stimulation, to proliferate, and to differentiate into cytotoxic effector cells. Humans receiving a supranutritional Se dose (200 μg/day) showed increased potential for cytotoxic T lymphocyte (CTL)-driven tumor lysis, mitogen-induced lymphocyte proliferation, and mixed lymphocyte reaction proliferation compared with a placebo group [85]. In both animals and humans, CD4^+^ T cells play a crucial in initiating immune responses, and antigen-specific CD4^+^ T cell proliferation and activation have been found to be increased in response to treatment with supranutritional amounts of Se [86]. Also, Se appears to abrogate the age-related deficiency of lymphocytes to respond to stimulation by proliferation and differentiation into cytotoxic effector cells [85]. However, the molecular mechanisms mediating these effects remain largely unexplored.

Because bone contains appreciable amounts of Se [87], it likely that Se plays a role in bone health. The hematopoietic system has been implicated as a primary target of Se at high doses [88], and osteoclasts are derived from hematopoietic progenitor cells of the monocyte-macrophage lineage [43]. Accordingly, selenite-treatment of osteoclast-like cells (e.g., RAW 264.7 cells) induced apoptosis as revealed by morphological changes, internucleosomal DNA fragmentation, activation of caspase-3, and generated the superoxide anion [28]. These findings indicate that selenite can induce apoptosis through the mitochondrial pathway in mature osteoclasts. 

Osteoclasts are known to be activated by inflammatory cytokines released at low levels by osteoblasts [30]. That Se can alleviate the NF-κB dependent regulation of the inflammatory response suggests that it may have a role in mediating the osteoblast-ostoclast crosstalk [44,45]. Recent data have shown that supplementation of osteoblasts with MSeA reduced the activation of NF-κB, leading to decrease in interleukin (IL)-6, monocyte chemoattractant protein-1 (MCP-1), cyclooxygenase-2 (COX-2) and inducible nitric oxide synthase (iNOS) [30]. Thus, it is likely that Se, at least at high doses, can prevent bone resorption through the inactivation of osteoclasts. Consistent with this observation, a recent study demonstrated that Se status was inversely related to bone turnover and positively correlated with BMD in healthy euthyroid postmenopausal women independent of thyroid status [8]. However, the other study indicated that elevated Se intake negatively affected bone mass measurements in postmenopausal women over the age of 51 but only if calcium intake was less than 800 mg/day [89]. Therefore, more future human studies are needed to determine the beneficial effect of Se on bone health.

## 4. Conclusions

Selenium is an essential nutrient that appears to play a role in bone health. That role is likely to involve the functions of selenoproteins. Many, if not all, selenoproteins are antioxidant enzymes that participate in maintaining cell redox balance, which is important in the regulation of inflammation and bone cell proliferation/differentiation. Selenium may play additional cellular roles, particularly at supranutritional doses, *i.e.*, doses greater than those required for maximal selenoprotein expression. These include the induction of cell cycle arrest, apoptosis, immune function, and the prevention of the bone resorption through the inactivation of osteoclasts (Figure 2). These cellular activities of Se at both nutritional and supranutritional doses may partially account for the potential protection against rheumatoid arthritis, osteoarthritis, osteoporosis and ROS produced by osteoclasts during bone remodeling [13,90,91,92].
